# Supplementary material for: The interactome of intact mitochondria by cross-linking mass spectrometry provides evidence for coexisting respiratory supercomplexes
Source: Mol Cell Proteomics. 2017 Dec 8;17(2):216–32. doi: 10.1074/mcp.RA117.000470 (PMC5795388; doi:10.1074/mcp.RA117.000470)
Supplement: Supplemental Data [file supp_RA117.000470_133922_0_supp_23978_fzffwf.zip › spectra_annotation/mito_spectra_annotation/310-1-6-1-26-1.pdf]

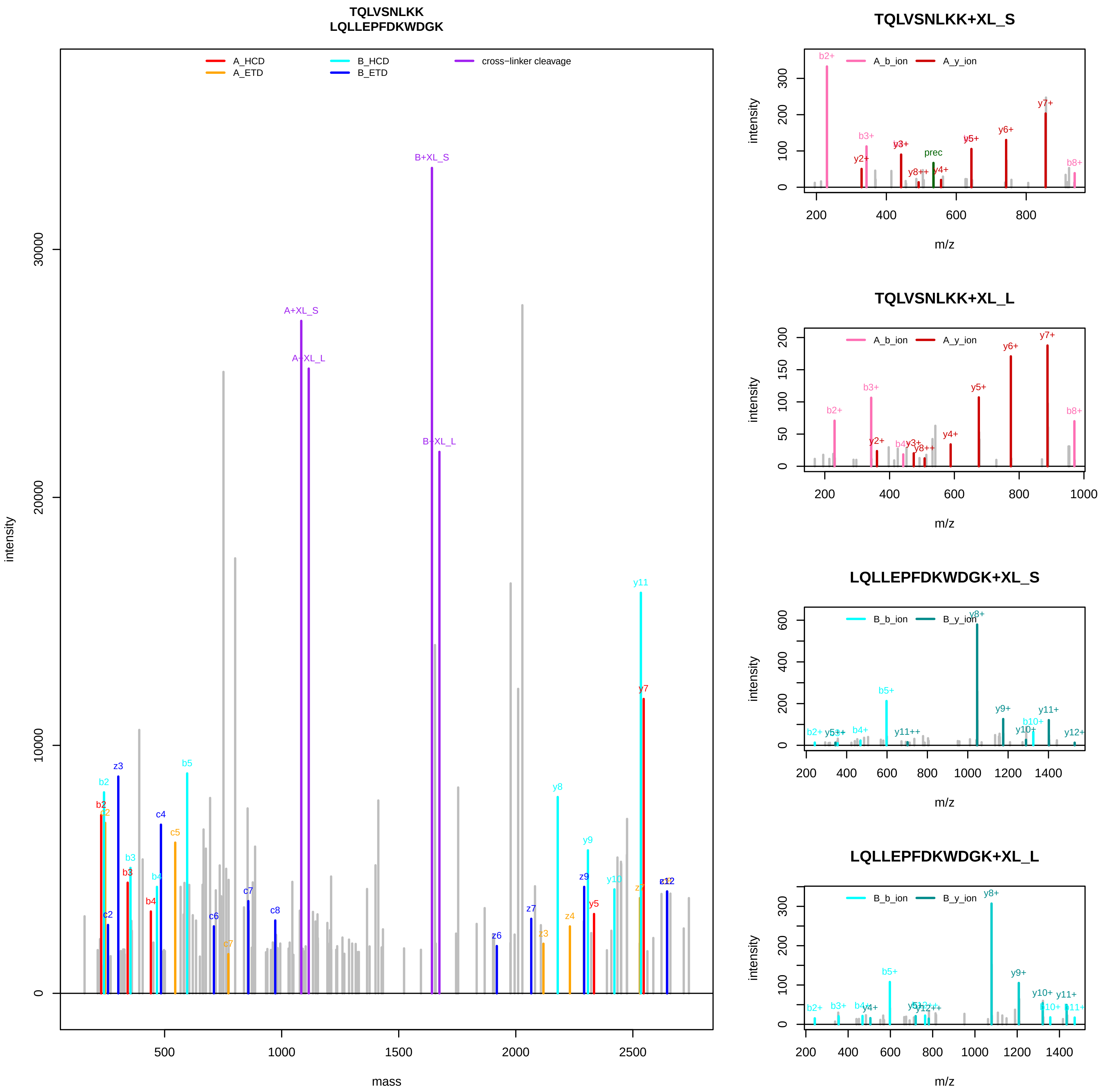
TQLVSNLKK
LQLLEPFDKWDGK
A_HCD
A_ETD
B_HCD
B_ETD
cross−linker cleavage
B+XL_S
B+XL_L
A+XL_S
A+XL_L
y11
y7
y8
y9
y10
z9
z7
z6
z3
z4
z7
z12
y5
b2
b3
b4
b5
c2
c3
c4
c5
c6
c7
c8
z3
intensity
mass
0
10000
20000
30000
500
1000
1500
2000
2500
TQLVSNLKK+XL_S
A_b_ion
A_y_ion
prec
intensity
m/z
TQLVSNLKK+XL_L
A_b_ion
A_y_ion
intensity
m/z
LQLLEPFDKWDGK+XL_S
B_b_ion
B_y_ion
intensity
m/z
LQLLEPFDKWDGK+XL_L
B_b_ion
B_y_ion
intensity
m/z
